# A polyphosphazene elastomer containing 2,2,2-trifluoroethoxy groups as a dielectric in electrically responsive soft actuators†

**DOI:** 10.1039/d4tc02369b

**Published:** 2024-07-11

**Authors:** Cansu Zeytun Karaman, Thulasinath Raman Venkatesan, Johannes von Szczepanski, Frank A. Nüesch, Dorina M. Opris

**Affiliations:** a Functional Polymers, Empa, Swiss Federal Laboratories forMaterials Science and Technology (EMPA) 8600 Duebendorf Switzerland dorina.opris@empa.ch; b Ecole Polytechnique Federale de Lausanne(EPFL) 1015 Lausanne Switzerland; c Eidgenössische Technische Hochschule Zürich (ETHZ) 8092 Zurich Switzerland

## Abstract

The adaptive structure and excellent actuation of dielectric elastomer actuators (DEAs) make them promising candidates for soft robotics, haptic interfaces and artificial muscles. A wide variety of elastomers have been synthesised and investigated as dielectrics. Inorganic polymers such as polysiloxanes and polyphosphazenes have a low glass transition temperature. While polydimethylsiloxane (PDMS) has made its way into DEAs, the latter has received little attention in this field. Here, we present a dielectric elastomer based on polyphosphazene modified with 2,2,2,-trifluoroethoxy groups as the dielectric, which exhibits a dielectric permittivity two times higher than polydimethylsiloxanes (PDMS), excellent elasticity and a high dielectric breakdown field. These properties enable fast, reliable actuation and higher electrostatic forces than conventional PDMS. The actuators can withstand repeated actuation cycles and are suitable for long-term reliability applications.

## Abstract 2

Dielectric elastomer actuators based on polyphosphazenes modified with trifluoroethoxy groups exhibit a dielectric permittivity two times higher than polydimethylsiloxanes (PDMS), excellent elasticity, and a high dielectric breakdown field.

## Graphical abstract

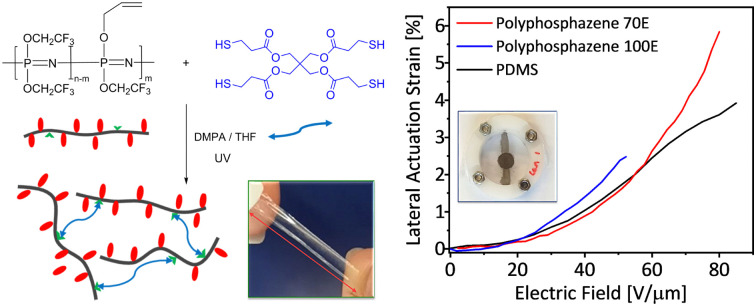

## Introduction

Elastomers are chemically or physically cross-linked polymers above their glass transition temperature (*T*_g_).^1^ These materials undergo deformation when subjected to mechanical stress. When appropriately cross-linked, they return to their original shape upon releasing stress. Smart elastomers can be specifically engineered to respond to various stimuli, including light, pH, temperature, as well as magnetic and electric fields.^2^ Due to their muscle-like actuation, these materials are often called artificial muscles and have received significant attention from the scientific community.^3^ Dielectric elastomer actuators (DEAs) are among the most promising artificial muscles.^4^ They can be easily constructed by assembling two stretchable electrodes on a dielectric elastomer to form an elastic capacitor. This capacitor is operated by charging/discharging it at a specific voltage and frequency. Its operation is silent, while the applied voltage precisely controls its actuation and force. Since the potential of these actuators was demonstrated by Pelrine *et al.*, many commercially available and designed elastomers with increased dielectric permittivity and reduced elastic modulus have been explored to improve their performance, and significant progress has been achieved.^5^ The most explored elastomers are based on polyacrylates, polyurethanes, poly(styrene-*co*-butadiene)s, and polysiloxanes.^6^ Thanks to their highly flexible backbone, polysiloxanes allow the achievement of polar elastomers after appropriate chemical modification with polar groups.^7,8^

Additionally, ultrasoft elastomers with very low elastic modulus have been reported. Such materials can be achieved by cross-linking bottlebrush polymers. The most investigated ones have a carbon–carbon backbone and polydimethylsiloxane (PDMS) brushes. Some were even used as a dielectric in DEAs with impressive performance.^9–11^ Most recently, a bottlebrush polymer elastomer having a polysiloxane backbone and polyphosphazene brushes has been reported, but its performance as a dielectric in actuators has not been investigated.^12^ Polyphosphazenes have gained tremendous attention due to their unique inorganic backbone, endowing them with exceptional properties distinct from their organic counterparts. These properties encompass biomedical compatibility, fire resistance, high flexibility, and gamma-radiation stability.^13^ Poly(organo)phosphazenes are organic–inorganic hybrid macromolecules with a backbone of alternating phosphorous–nitrogen single and double bonds with two organic side groups attached to the phosphorous atom.^14^ Despite Stokes's early synthesis in 1897, this field gained importance only after Allcock *et al.* showed that they could be made soluble.^15^

Polyphosphazenes allow for chemical modification by a large variety of substituents, making them electro, optically, light, and biologically active, resistant to solvent and aggressive chemicals, and ionically conductive.^16^ However, their potential as a dielectric in DEAs received little attention despite their potential.^17^ Polyphosphazenes combining fire resistance, increased dielectric permittivity, and tuneable mechanical properties are attractive for DEAs applications.

Polyphosphazene modified with 2,2,2-trifluoroethoxy groups is known to have a low *T*_g_ and increased dielectric permittivity, thus meeting critical material properties for a dielectric elastomer.^18^ Here, we report polyphosphazene-based elastomers modified with trifluoroethoxy groups as dielectrics in actuators. We introduced 2,2,2-trifluoroethoxy groups to increase the dielectric permittivity and a small number of allyl groups to cross-link the polyphosphazene *via* a thiol–ene reaction with a multifunctional thiol. Elastic materials were achieved, and their dielectric, mechanical, and electromechanical properties were evaluated and compared with Elastosil, a well-known PDMS elastomer currently used in commercial DEAs.^19^

## Results and discussion

**Fig. 1:**
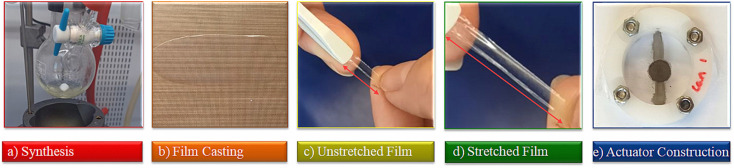
Steps involved in achieving a dielectric elastomer actuator with a polyphosphazene-based elastomer as a dielectric: (a) synthesis of the polar polyphosphazene; (b) thin film formation and cross-linking to transparent films; (c) and (d) testing the mechanical properties of the elastomer; (e) constructing and testing of actuators.

The procedure involves the synthesis of polychlorophosphazene in sufficiently large quantities and its functionalization with 2,2,2-trifluoroethoxy about 5 mol% of allyloxy groups. Following this, thin films were formed and cross-linked, and the mechanical properties were optimized to meet the requirements of a dielectric in actuators (Fig. 1). The synthesis of the starting polydichlorophosphazene, which is commercial but very expensive, caused unexpected problems, which are described in the Experimental section. The living cationic condensation polymerization of trichloro(trimethylsilyl)phosphoranimine (Cl_3_P=NSiMe_3_) in the presence of PCl_5_ catalyst described by Allcock, Manners, and Honeyman was easier to reproduce in our lab using a regular Schlenk line setup.^20^ Unlike the other routes, this polymerization reaction occurs at room temperature. Besides, high molecular weight polymers with narrow distributions can be obtained by adjusting the monomer-to-initiator ratio. Niecke and Bitter first reported the synthesis of Cl_3_P=NSiMe_3_ monomer.^21^ They reacted lithium bis(trimethylsilyl)amide (LiN(SiMe_3_)_2_) with phosphorus pentachloride (PCl_5_) at 10 °C under inert conditions, but the reported yield was rather poor. Manners *et al.* suggested decreasing the reaction temperature to −78 °C and using less reactive PCl_3_ instead of PCl_5_ to obtain the intermediate Cl_2_PN(SiMe_3_)_2_.^22^ Sulfuryl chloride subsequently oxidized this intermediate to Cl_3_P=NSiMe_3_ in 80% yield. Although this synthetic path allowed us to achieve the desired product, the inclusion of an additional reaction step employing highly toxic sulfuryl chloride, combined with the likelihood of Cl_3_P=NSiMe_3_ decomposition into hexachlorocyclophosphazene during synthesis and distillation, prompts us to explore alternative synthetic routes.

**Scheme 1:**
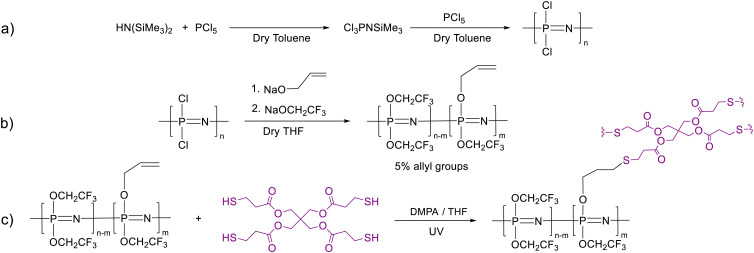
Synthesis of the monomer starting from hexamethyldisilazane and PCl_5_ and its polymerization using a PCl_5_ initiator (a); functionalization with trifluoroethoxide and 5 mol% allyl alkoxide groups (b); and cross-linking with pentaerythritol tetrakis(3-mercapto propionate) *via* a thiol–ene reaction under UV light using DMPA initiator (c).

With some small modifications, the synthetic path starting from 1,1,1,3,3,3-hexamethyldisilazane (HMDS) and PCl_5_ was found to work best in our lab (Scheme 1a). This synthesis has been described before by Kireev *et al.* and further optimized by Hacivelioglu *et al.*^23,24^ We have found that adding PCl_5_ at once at −84 °C prevents the reaction temperature from reaching above room temperature and forming (NPCl_2_)_3_ as well as short chains. ^31^P NMR shows the characteristic signal for the monomer at −55.4 ppm with some minor contamination with hexachlorocyclophosphazene (20.5 ppm) (Fig. S1a, ESI†). The polymerization was initiated by PCl_5_, whereby the monomer-to-initiator ratio was kept constant at 800 : 1. After overnight mixing, the polymer solution was filtered under argon, and the solvent was distilled at 3 mbar. The ^31^P NMR spectrum shows the typical signal at −18.3 ppm for polydichlorophosphazene and a small peak at 19.9 ppm for the cyclic monomer, representing about 6.5 wt% (Fig. S1b, ESI†). Nonetheless, it remains ambiguous whether the cyclic monomer forms during synthesis or arises during NMR measurements, where it might occur due to an increase in temperature during sample preparation and measurement.

**Fig. 2:**
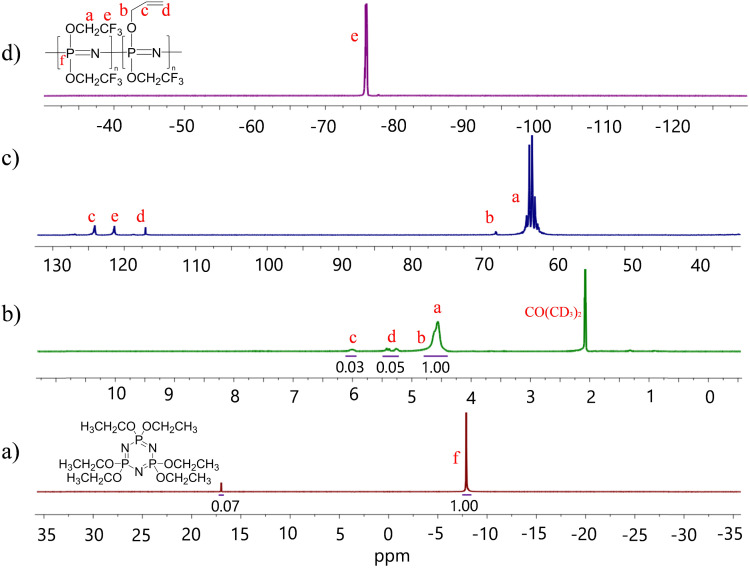
^31^P (a); ^1^H (b); ^13^C (c); ^19^F NMR (d) spectra of the allyl alkoxide substituted poly[bis(2,2,2-trifluoroethoxy)phosphazene] in (CD_3_)_2_CO at room temperature.

The polydichlorophosphazene was dissolved in dry THF to replace the chloride with the desired substituents by nucleophilic substitution (Scheme 1b). It should be mentioned that after dissolving the polymer in THF, the nucleophile should be added rather fast to prevent THF polymerization. An alternative approach is to use dioxane, which performs equally well as THF in this reaction but does not undergo cationic polymerization. For the nucleophilic substitution, we first reacted about 5% of the chlorine atoms with allyl groups using allyl alkoxide (Fig. S1c, ESI†), followed by the substitution reaction with sodium 2,2,2-trifluoroethoxide to consume all chlorine. The effectiveness of substitution is evidenced by ^31^P, ^1^H, ^13^C, and ^19^F NMR spectra (Fig. 2). The characteristic ^31^P NMR signal at −18.3 ppm for polydichlorophosphazene shifted to −7.8 ppm for the allyloxy and trifluoroethoxy substituented polyphosphazene, indicating successful substitution (Fig. 2a). Additionally, the ^1^H NMR data (Fig. 2b) shows that about 5 mol% of allyl groups were introduced. ^13^C NMR spectrum confirms the nucleophilic substitution and the ^19^F NMR spectrum confirms the presence of trifluoroethoxy groups (Fig. 2c and d). Achieving a quantitative substitution reaction is essential as any unreacted chlorine will undergo hydrolysis over time, leading to a slow alteration in the mechanical properties of the polymers. However, quantifying traces of unreacted chlorine is challenging.^25^

The formed polymer was isolated as a transparent viscous liquid. GPC investigation reveals a number average molecular weight M_*n*_ of 71 926 g mol^−1^ and a dispersity of 2.07 (Fig. S2 and Table S1, ESI†). To form elastic materials, the allyl groups of the polymer were utilized in a UV-induced thiol–ene reaction with pentaerythritol tetrakis(3-mercaptopropionate) cross-linker (Scheme 1c). IR investigations support the presence of the expected ester and thioether groups (Fig. S3, ESI†). To optimize the mechanical properties, we tuned the amount of cross-linker. Thus, a series of elastomer materials E*x* were synthesized, where *x* represents the volume in μL of the cross-linker solution used per g polymer (E50, E70, and E100). For the amount of reagents used, please refer to Table 1.

**Table 1:** Amount of reagents used for cross-linking polyphosphazene (1 g) and the strain at break, Young modulus at 10% strain, storage modulus (*E*′), and loss tangent tan (*δ*)

Materials	CL [mmol]^a^	DMPA [mg]	*s* [%]	*Y*_10%_ b [kPa]	*E*′^ ^c [MPa]	tan (*δ*)^c^
E50	0.131	5	—	—	—	—
E70	0.183	5	289 ± 2	294 ± 40	366 ± 10	0.099 ± 0.009
E100	0.262	5	171 ± 62	690 ± 11	700 ± 230	0.081 ± 0.008
Elastosil	—	—	224 ± 42	1450 ± 77	1.26 ± 0.01	0.031 ± 0.001

a A solution of pentaerythritol tetrakis(3-mercaptopropionate) CL (400 μL) in THF (2000 μL) was used.

b *Y*_10%_ was taken as the tangent to the stress–strain curves using a linear fit from 0 to 10% strain.

c Storage modulus (*E'*) and tan (*δ*) at 1 Hz.

**Fig. 3:**
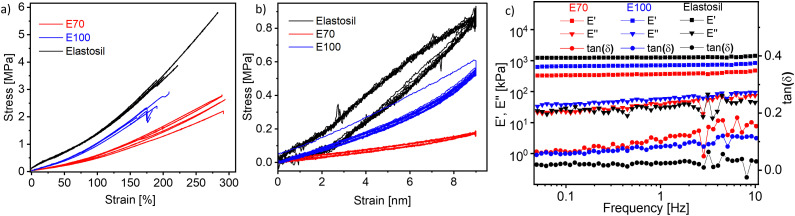
Stress–strain curves of E70, E100, and Elastosil (a). For each material, at least five specimens were tested. Overview of the ten cycles of uniaxial cyclic tensile curves at strains between 0 and 50% E70, E100, and Elastosil (b). Results for dynamic-mechanical analysis (DMA) of the films E70 and E100 (c) at different frequencies, 2% strain, and room temperature.

E50 was only partially cross-linked; therefore, no characterizations were conducted on this material. However, materials E70 and E100, for which more cross-linker was used, were elastic. Uniaxial and cyclic tensile tests were performed to evaluate the elastic performance (Fig. 3). Five samples were measured and compared with an Elastosil film, a commercial PDMS-based elastomer well known to the DEA community. The Young's modulus of the materials was determined as the slope of the stress–strain curve from 0 to 10% strain. Their elastic modulus was 294 ± 40 kPa for E70, 690 ± 11 kPa for E100, and 1450 ± 77 MPa for Elastosil, respectively. E100 is stiffer than E70, which aligns with the increasing cross-linker amount. Both materials have good strain at break of 289 ± 2% for E70 and 171 ± 62% for E100, respectively.

Cyclic tensile tests were conducted for the hysteresis test. Each sample was subjected to cyclical loading (50% strain) and unloading for 10 cycles for 30 seconds at each load and unload state (Fig. 3b). Large hysteresis between the first load and unload cycle was observed for the stiffest material E100. However, in the subsequent cycles, the hysteresis was rather small and the material recovered its initial length. For E70, the hysteresis is negligible, confirming the good elastic properties of our material.

Thermogravimetric analysis shows that both materials were stable up to 200 °C, at which point they started to decompose (Fig. S4, ESI†).

Dynamic mechanical analysis was conducted to determine the storage modulus (*E*′), loss modulus (*E*′′), and loss tangent (tan (*δ*)) at room temperature by altering frequency while applying 2% strain (Fig. 3c). Generally, there is a small increase in the storage modulus with increasing frequency for both materials as well as for Elastosil. The storage modulus at 1 Hz increases from 366 kPa for E70 to 700 kPa for E100 with increasing cross-linker concentration and only slightly increases with frequencies. Elastosil is stiffer than both polyphosphazene elastomers and shows a storage modulus of 1.26 MPa. The mechanical losses of the two materials at low frequencies are below 0.1 at 1 Hz and below 0.2 at 10 Hz, thus confirming the good elasticity of our materials. However, both E70 and E100 materials showed slightly higher mechanical losses than Elastosil. Low mechanical losses are important in actuators operated at high frequencies, allowing fast and reversible actuation. Because material E70 was about two times softer than E100, this material was selected for further investigation.

The outstanding elasticity of polybis(trifluoroethoxide)phosphazene is due to the molecular structure of this polymer, which has a highly flexible backbone consisting of alternating phosphorus and nitrogen atoms.^26^ The interactions between the polymer chains also play an important role in determining the elasticity of the material. The trifluoroethoxide groups have intermolecular dipole–dipole interactions that contribute to the improved elasticity of the polymer.^27^ Finally, the degree of cross-linking within the polymer network has been carefully controlled to tailor the mechanical properties for DEA applications.^28^

**Fig. 4:**
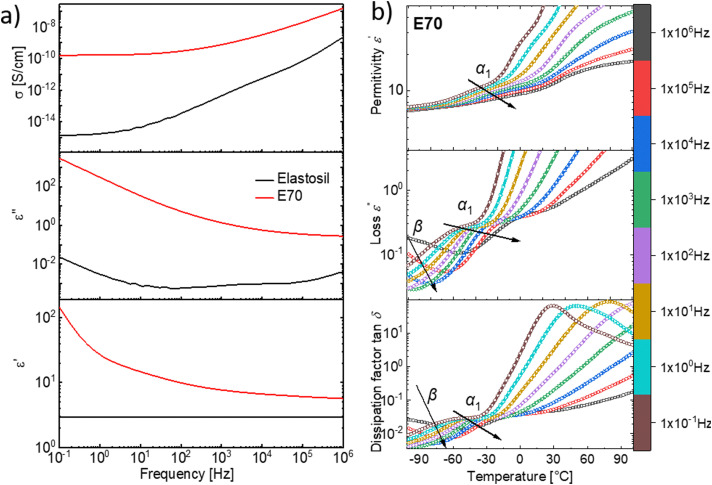
The conductivity (*σ*′), loss tangent (tan (*δ*)), dielectric loss (*ε*′′), and dielectric permittivity (*ε*′) at room temperature as a function of frequency ranging from 10^−1^ and 10^6^ Hz of E70 and Elastosil (a) and dielectric permittivity, permittivity loss, and dissipation factor tan (*δ*) of E70 from −100 to 100 °C at different frequencies (b).

Dielectric impedance spectroscopy provides information about the polarizability of the elastomers as a function of frequency. The dielectric response of materials is measured by applying a small alternating voltage (1 V) at different frequencies (Fig. 4a).^29^ The conductivity (*σ*′), dielectric loss tangent (tan (*δ*)), dielectric loss (*ε*′′), and relative permittivity (*ε*′) of E70 at room temperature are plotted as a function of frequency. The conductivity of E70 is quite low (≈10^−10^ S cm^−1^) even though polyphosphazene has alternating single and double bonds in its backbone. This emerges from the interruption of conjugation due to a mismatch of the 3d orbital of phosphorus and the 2p orbital of nitrogen. Besides, the trifluoro ethoxide side group favors the *trans*–*trans* conformation to decrease structural repulsions, leading to the localized conjugation in their skeleton.^30–33^

The dielectric permittivity is 5.65 at 10^6^ Hz at room temperature for material E70, while the dielectric permittivity of Elastosil is about two times lower at the same frequency. The dielectric permittivity of E70 is only slightly lower than the one of trifluoropropyl modified silicone elastomers, with 58% siloxy repeat units containing trifluoropropyl groups with *ε*′ = 6.4.^34^ Assuming the two polymers have the same density, the concentration of CF_3_ groups in the fluorinated silicone (0.48 mol CF_3_ in 100 g) is lower than in the modified polyphosphazene (0.82 mol CF_3_ in 100 g), however, their permittivity is similar.^34^ This indicates that the polar groups in polyphosphazene cannot be polarized as effectively as in polysiloxanes. It is known that polyphosphazene modified with trifluoroethoxy groups is semicrystalline, which may explain the somewhat lower-than-expected value for the permittivity. However, no evidence of crystalline structure has been found from the impedance and DSC measurements performed in this work (Fig. S5, ESI†). Presumably, the introduction of chemical cross-links prevents crystallization.^35^

**Fig. 5:**
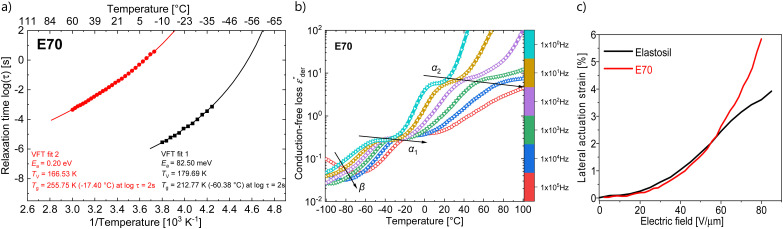
Arrhenius relaxation plot with VFT fits for samples E70 (a); isochronal representation of the conduction-free dielectric loss 
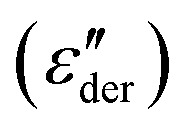
 calculated for E70 sample between 10^−1^ Hz and 10^6^ Hz (b), and lateral actuation strain of E70 and Elastosil at different electric fields (c).

Temperature-dependent dielectric impedance measurements can reveal the various relaxation processes occurring in the material. The plot of dielectric permittivity, along with its losses and dissipation factor measured between −100 to +100 °C for E70 are plotted in Fig. 4b. Starting from a low temperature, around −100 °C, we see traces of relaxation in the dielectric loss data, which could be assigned to *β* relaxations^36,37^ that arise due to the local molecular motion of the alkoxy side groups. Above this temperature, we observe another set of loss peaks shifting to higher temperatures with increased frequency. Similar processes were observed for material E100 (Fig. S6, ESI†). Previous dielectric studies on similar alkoxy-substituted polyphosphazene assigned this relaxation to the glass-transition relaxation process due to unfreezing the –P=N– backbone.^37–40^ By fitting the loss peaks with the well-known Havriliak–Negami (HN) function using the DCALC program developed by Wübbenhorst *et al.*,^41,42^ a classical Vogel–Fulcher–Tammann (VFT) fit was obtained as plotted in Fig. 5a with a calculated *T*_g_ of −60.4 °C for E70 at a relaxation time of 100 s (log *τ* = 2 s).^43^ The calculated values closely agree with those found in the literature.^37,40^

Above 0 °C, a steep increase in the dielectric permittivity and dielectric losses are observed. This can be attributed to the contribution of ionic conductivity due to the increase in mobility of the ionic species in the samples above their *T*_g_. They manifest as peaks in the dissipation factor tan *δ* plot above 0 °C and at low frequencies, as seen in Fig. 5b. From previous reports, in addition to the *T*_g_, two additional transitions have been observed in alkoxy-substituted polyphosphazene owing to their semicrystalline nature.^37–40^ This includes the melting point of the crystals (*T*_m_) and an intermediate transition from the semicrystalline state to the mesomorphic state (*T*_1_). As mentioned above, such transitions were not observed in the impedance and DSC measurements. However, it should be noted that the crystallization of polymers is a slow time-dependent process.

Though we do not observe the presence of additional structural transitions *T*_1_ and *T*_m_, evidence of a further transition showing frequency-independent behavior is revealed when the dielectric impedance data is subjected to a derivative analysis (conduction-free dielectric loss derivative; 
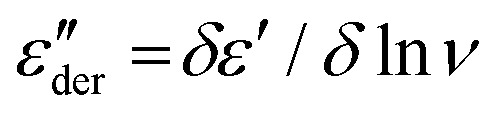
)^41,42^ as shown in Fig. 5b. By HN-peak fitting, an Arrhenius plot is obtained and the VFT fit to the data is plotted in Fig. 5a (VFT fit 2). Hence, this process is referred to as the *α*_2_ relaxation. The presence of two *α* relaxations is unexpected. The origin of this second glass transition at −17.4 °C might point to the absorption of water molecules by the polymer. Water in our polymer network may also explain the slow degradation through hydrolysis of the network when kept under normal environmental conditions. Such hydrolysis processes have been observed before, however, further investigations are needed to confirm them.^44^

**Fig. 6:**
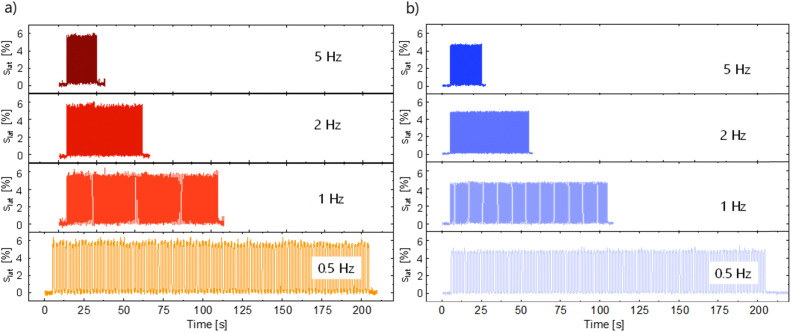
Lateral actuation strain of E70 over 100 actuation cycles at 3600 V (80 V μm^−1^) (a) and Elastosil at 3640 V (85 V μm^−1^) at frequencies from 0.5 Hz to 5 Hz (b).

Dielectric elastomer actuators were constructed by placing a 5% pre-strained thin film between two rigid circular frames (Fig. 1e). Two electrodes of carbon black powder with a diameter of 8 mm were applied on both sides of the dielectric film. We gradually increased the voltage until dielectric breakdown was reached, while the lateral actuation strain was measured with a camera. The electromechanical response of E70 was compared with the well-known silicone elastomer Elastosil, which has been widely explored in DEAs (Fig. 5c). The highest actuation strain of 5.8% at 80 V μm^−1^ was measured for E70 (45 μm thick film), while Elastosil gave 3.9% lateral actuation at 85 V μm^−1^ (20 μm thick film). The stability of the actuation of E70 was tested for 100 cycles at 3.6 kV, revealing a stable actuation up to a frequency of 5 Hz, the maximum frequency investigated (Fig. 6). No hysteresis was observed, confirming the developed elastomer's good elastic properties. Additionally, the actuation exhibits the same reversibility as regular PDMS. Also, material E100 exhibits reversible actuation, but its response was inferior to the one observed for E70, as E100 was stiffer (Fig. S7–S9, ESI†). It should be noted that previous investigations on the actuation behavior of polar silicone elastomers show that if the *T*_g_ is not significantly lower than room temperature, a strong frequency dependence behavior was observed.^45^ These experiments on polyphosphazene elastomers confirm that the *T*_g_ of −60.4 °C is sufficiently low, so the actuation remained constant at frequencies between 0.5 and 5 Hz. The electrostatic pressure *p* acting on the films is given by the Maxwell pressure *p* = *ε*′*ε*_0_*E*^2^, where *ε*_0_ is the permittivity of vacuum, *ε*′ is the relative permittivity of the material, and *E* is the applied electric field. The electrostatic pressure is 0.32 MPa for E70 and 0.17 MPa for Elastosil at an electric field of 80 V μm^−1^. Thus, the actuation pressure of E70 is about two times higher than that of Elastosil at the same electric field.

Weibull probability plots were also used to analyze the dielectric breakdown (Fig. S10, ESI†). These plots offer insights into the statistical distribution of breakdown strengths. As depicted in the plots, our materials exhibit a high probability of breakdown above 50%. Furthermore, the average breakdown field of the two materials was between 75 and 80 V μm^−1^. Additionally, the cyclic tests show that our materials (E70 and E100) can be cycled for more than 100 cycles, comparable to nitrile-butadiene rubber (NBR) and VHB film, suggesting that our materials can compete favorably with commercially available materials.^46,47^ Furthermore, our materials can reversibly respond to higher frequencies compared to NBR and VHB.^48,49^

## Conclusions

Polybis(trifluoroethoxide)phosphazene with 5% allyl alkoxide side group was successfully synthesized on the 20 g-scale and cross-linked into homogeneous, transparent films with thicknesses of 50 μm. The materials have excellent mechanical properties with low viscous losses, exhibiting an almost linear storage modulus at varying frequencies. Dielectric characterization revealed good insulator properties and a room-temperature dielectric permittivity of 5.65 at 1 MHz. Frequency-dependent dielectric measurements revealed that these materials have a *T*_g_ of −60.4 °C, significantly below room temperature and thus highly attractive for outdoor applications. Actuators exhibited stable and reversible actuation even at 5 Hz for 100 cycles with no hysteresis. Besides, for an E70 sample, an actuation strain of 5.83% at 80 V μm^−1^ was recorded, which is higher than the one of PDMS at the same electric field. In conclusion, the increased dielectric permittivity and the high electric fields these materials can withstand, together with the excellent film formation capabilities and mechanical properties, make polyphosphazene elastomers an attractive candidate for DEAs. Additionally, the large-scale synthesis of polyphosphazene modified with 2,2,2-trifluoroethoxy groups demonstrates the scalability of production of this modified polymer, highlighting its potential in DEAs. Moreover, the polyphosphazene elastomers synthesised have two identical groups on the repeat unit, which facilitates crystallization. This feature can be critical in achieving certain properties, such as piezoelectricity.

## Experimental

### Materials

Unless otherwise mentioned, all chemicals were used without further purification. The reactions were carried out under an argon atmosphere with Schlenk setup. Allyl alcohol, phosphorus pentachloride, NaH (60% suspension in mineral oil), 2,2-dimethoxy-2-phenylacetophenone (DMPA), and pentaerythritol tetrakis(3-mercapto propionate) were purchased from Sigma-Merck.

1,1,1,3,3,3-Hexamethyldisilazane (HMDS) was purchased from ABCR and distilled before use. 2,2,2-Trifluoroethanol was purchased from CHEMOS Gmbh. 2,2,2-Trifluoroethanol and allyl alcohol were distilled over CaH_2_ under an argon atmosphere before use. Toluene and THF were purchased from VWR and dried over sodium/benzophenone before use. The Cl_3_PNSiMe_3_ monomer and polydichlorophosphazene were synthesized according to the literature with slight modifications.^24^

### Characterization

^1^H, ^13^C, ^19^F and ^31^P NMR spectra were recorded on a Bruker AV-III 400 spectrometer (Bruker BioSpin AG, Switzerland) using a 5 mm CryoProbe™ Prodigy probe at 400.2, 100.6, 376.5 and 162.0 MHz, respectively. All NMR experiments were performed at 298 K using the Bruker standard pulse programs and parameter sets. ^1^H and ^13^C NMR chemical shifts (*δ*) were calibrated to residual solvent peaks and ^19^F and ^31^P NMR chemical shifts were referenced to external samples with neat CFCl_3_ and H_3_PO_4_ at 0.0 ppm, respectively. For the water-sensitive compounds, the deuterated solvent was dried. The NMR spectrum of the Cl_3_PNSiMe_3_ monomer and polydichlorophosphazene were recorded in dry CDCl_3_ and the ones of poly[bis(2,2,2-trifluoroethoxy)]phosphazene in (CD_3_)_2_CO.

The tensile and cyclic uniaxial tensile stress tests were performed on a Zwick Z010 test machine with a crosshead speed of 50 mm min^−1^. Tensile test specimens with a gauge width of 2 mm and a gauge length of 18 mm were prepared by die-cutting. The strain was determined over the traverse moving sensor. The curves were averaged from five different samples per material using Origin software. The tensile modulus was determined from the slope of the stress–strain curves using a linear fit to the data points up to 10% strain.

Dielectric permittivity measurements were carried out using a Novocontrol Alpha dielectric analyzer, and the sample temperature was controlled using a Novocontrol Quatro cryosystem under a dry nitrogen atmosphere. The samples were prepared by placing uniform films between two compliant stainless steel electrodes with a diameter of 20 mm. The dielectric permittivity of the materials was analyzed within a frequency ranging from 10^−1^ to 10^6^ Hz.

Dynamic mechanical measurements were recorded on a RSA 3 DMA from TA Instruments. Stripes with a width of 10 mm and a length 25 mm were analyzed under 2.5 g dynamic load, at 2% strain, with the frequency ranging from 0.05 to 10 Hz at 25 °C.

TGA was conducted using a PerkinElmer TGA7 with a heating rate of 10 °C min^−1^ under a nitrogen gas flow from 25 °C up to 600 °C.

### Synthesis of monomer Cl_3_PNSiMe_3_^24^

Freshly distilled HMDS (18.00 g, 23.38 mL, 111.53 mmol) and dry toluene (100 mL) were added to a 250 mL Schlenk flask with a magnetic stirrer bar under an inert atmosphere. The reaction flask was placed into a −84 °C cold bath using a mixture of ethyl acetate/liquid N_2_. The flask was left to stand for 15 min to reach a stable temperature. Then, PCl_5_ (17.42 g, 83.65 mmol) was added at once and the reaction was stirred at this temperature for an hour. The desired product was confirmed by ^31^P NMR (162 MHz, CDCl_3_) *δ* = −55.4 ppm.

### Failed attempts to synthesize polydichlorophosphazene^24^

A seemingly convenient way to synthesize this polymer involves ring-opening polymerization (ROP) of ultrapure hexachlorocyclophosphazene monomer. This polymerization requires a sealed glass technique and specially designed setups to allow stirring. Additionally, the high temperature needed, the relatively large amount of unreacted cyclic monomer, and the possibility of failure due to unwanted cross-linking discouraged us from further exploring this synthetic path.^50^

The literature also reported the solution ring-opening polymerization of (NPCl_2_)_3_ as a way to synthesize polyphosphazene. It occurs in the presence of H_2_NSO_3_H catalyst with CaSO_4_ × 2H_2_O promoter and a high boiling solvent such as 1,2,4-trichlorobenzene (b.p.= 214 °C). Nevertheless, carefully adjusting the promoter amount is important to avoid cross-linking.^51^

The other synthetic route includes the reaction of ammonium chloride (NH_4_Cl) with phosphorus pentachloride (PCl_5_) at 150 °C. This is the main reaction to synthesize cyclic phosphazenes like (NPCl_2_)_3_ and (NPCl_2_)_4_. Therefore, NH_4_Cl to PCl_5_ ratio should be tuned carefully to obtain long-chain polychlorophosphazene. Nevertheless, the polymer has broad molecular weight distribution and lower molecular weight than the one synthesized by ROP.^50^

The condensation polymerization proposed by Emsley and De Jaeger starting from (NH_4_)_2_SO_4_ and PCl_5_ at 165 °C with the formation of Cl_2_P(O)–N=PCl_3_ phosphoranimines monomer requires temperatures between 230 and 290 °C for the initiation. Additionally, phosphoryl trichloride P(O)–Cl_3_ is formed as a side product, which is toxic. Therefore also, this method became unattractive.^52–54^

### Synthesis of polydichlorophosphazene^24^

To the *in situ* Cl_3_PNSiMe_3_ monomer solution synthesized above, PCl_5_ (21.77 mg, 104.56 μmol) was added at once at a −15 °C cold bath prepared by mixing ice/water and NaCl. The monomer-to-initiator mole ratio of 800 : 1 (Cl_3_PNSiMe_3_ : PCl_5_ = 83.64 mmol : 104.56 μmol) was kept constant for all experiments. The reaction mixture was stirred overnight (about 16 h) under an argon atmosphere and let to warm to room temperature. After the polymerization, the white turbid reaction mixture was filtered under argon to remove ammonium chloride. The filtrate was distilled at 3 mbar at room temperature to remove ClSiMe_3_ and the solvent.

### Synthesis of allyl alkoxide functionalized poly[bis(2,2,2-trifluoroethoxy)]phosphazene^55^

The polymer (9.86 g, 83.63 mmol) was dissolved in dry THF (100 mL). Then, a freshly prepared sodium allyl alkoxide (1.01 g, 12.65 mmol) solution in dry THF (50 mL) was added dropwise to the polymer solution under an inert atmosphere. The reaction mixture was stirred at reflux for 3 h. After cooling reaction to room temperature, sodium 2,2,2-trifluoroethoxide (16.88 g, 0.18 mol) solution in dry THF (100 mL) was added dropwise under an inert atmosphere, and then the mixture was stirred at reflux for 2 days. The reaction mixture was concentrated at reduced pressure and the resulting slurry was precipitated three times in diluted HCl (10% in water) solution to pH = 7. Then, the polymer was dissolved in acetone and washed with MeOH to obtain the desired polymer in 85% yield. The desired product was confirmed by ^1^H NMR (400 MHz, (CD_3_)_2_CO) *δ* = 4.6 ppm, ^13^C NMR (100 MHz, (CD_3_)_2_CO) *δ* = 63.02 ppm (–CH_2_), 125.47 (–CF_3_), ^31^P NMR (162 MHz, (CD_3_)_2_CO) *δ* = −7.81 ppm, ^19^F NMR (376 MHz, (CD_3_)_2_CO) *δ* = −77.6 ppm. For 5% allyl alkoxy group; ^1^H NMR (400 MHz, (CD_3_)_2_CO) *δ* = 4.81 ppm (–CH_2_–), 6.01 ppm (–CH=), 5.17–5.48 ppm (=CH_2_), ^13^C NMR (100 MHz, (CD_3_)_2_CO) *δ* = 68.04 ppm (–CH_2_–), 132.56 (–CH=), 117.32 (=CH_2_) ^31^P NMR (162 MHz, (CD_3_)_2_CO) *δ* = −7.71 ppm.

### Cross-linker solution preparation

Pentaerythritol tetrakis(3-mercaptopropionate) (0.512 g, 400 μL) was weighed in a vial and THF (2000 μL) was added to give a solution of 1 : 5 (v : v) of cross-linker in THF. Each elastomer was produced by using a freshly prepared cross-linker solution in a brown bottle.

### Thin film formation

A homogenous solution of polymer (1 g), cross-linker (100 μL, 70 μL, or 50 μL), DMPA initiator (5 mg) in minimum THF (1 mL) was made using a speed mixer for 5 min at 3000 rpm. The mixture was blade coated (blade thickness 100 μm) on a Teflon substrate and left overnight in a laminar flow to allow the solvent to evaporate. It was then irradiated with UV light for 5 min to give a cross-linked elastic film E*x*, where *x* represents the volume of cross-linker solution used per g polymer, *e.g.* 100 μL cross-linker solution in THF (1 : 5 v : v) for E100, 70 μL for E70, and 50 μL for E50, respectively. The cross-linked films were kept in a vacuum oven at 60 °C for 1 day before use.

### Actuator construction

The film was biaxially prestrained by 5%. The prestrain in the film was kept by fixing the film between two circular rigid plastic frames. Carbon black powder circular electrodes with a diameter of about 8 mm were applied on both sides and connected to a high-voltage source using two Al stripes. The actuation strain was measured optically as the extension of the diameter of the electrode area *via* a digital camera, using an edge detection tool of a LabView program to detect the boundary between the black electrode area and the transparent silicone film.

## Author contributions

C. Z. K. performed the synthesis of all polymers and materials and the characterization of polymers. T. R. V. conducted the impedance spectroscopy investigations. J. S. conducted the DMA measurements, C. Z. K. conducted the tensile test measurements. D. M. O. conducted actuator measurements. C. Z. K. and D. M. O. wrote the original draft. D. M. O. initiated the activity, designed the materials, received funding acquisition, and coordinated and supervised this research. All authors contributed with discussions, reviewing, and editing and have approved the final version of the manuscript.

## Data availability

Raw data for the paper can be found: https://doi.org/10.5281/zenodo.11499705.

## Conflicts of interest

There are no conflicts to declare.

## Supplementary Material

TC-012-D4TC02369B-s001
