# Supplementary material for: A polyphosphazene elastomer containing 2,2,2-trifluoroethoxy groups as a dielectric in electrically responsive soft actuators
Source: J Mater Chem C Mater. 2024 Jul 11;12(33):12825–34. doi: 10.1039/d4tc02369b (PMC11340341; doi:10.1039/d4tc02369b)
Supplement: TC-012-D4TC02369B-s001 [file TC-012-D4TC02369B-s001.pdf]

Electronic Supplementary Material (ESI) for Journal of Materials Chemistry C.
This journal is © The Royal Society of Chemistry 2024

**Supporting Information**

**A polyphosphazene elastomer containing 2,2,2-trifluoroethoxy groups as a dielectric in electrically responsive soft actuators**

*Cansu Zeytun Karaman[a,b], Thulasinath Raman Venkatesan[a], Johannes von Szczepanski[a,c], Frank A. Nüesch[a,b], and Dorina M. Opris[a,c]**

[a]*Functional Polymers,Empa, Swiss Federal Laboratories forMaterials Science and Technology (EMPA), 8600 Duebendorf,Switzerland.*

[b]*Ecole Polytechnique Federale de Lausanne(EPFL), 1015 Lausanne, Switzerland*

[c]*Eidgenössische Technische Hochschule Zürich (ETHZ) ), 8092 Zurich, Switzerland*

E-mail: dorina.opris@empa.ch

# Contents

NMR Spectra ........ 2

GPC Spectrum ........ 2

FTIR Spectra ........ 3

DSC Spectra ........ 4

TGA Spectra ........ 4

Dielectric Spectra ........ 5

Actuation Measurements ........ 5

Weibull probability plot ........ 8

Calculation of electrostatic pressure ........ 8

References ........ 8

## NMR Spectra

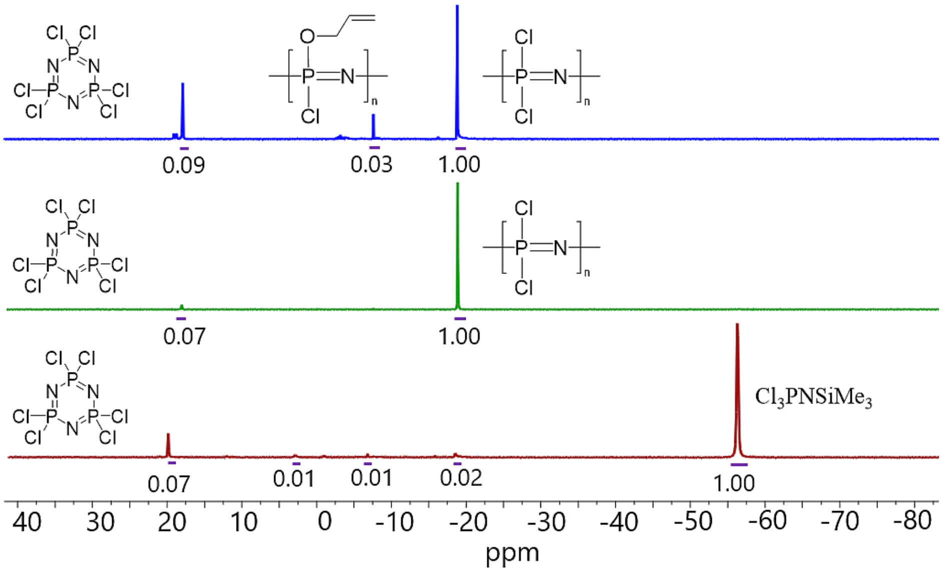


**Figure S1.** $^{31}P\{^{1}H\}$ NMR spectra ($CDCl_3$) of monomer $Cl_3PNSiMe_3$ with cyclohexachlorotriphosphazene (0.7 %<) and some oligomer impurities (0.2 %<) (a); polymer with cyclohexachlorotriphosphazene (0.7 %<) impurity (b); polymer after allyl alkoxide substitution (c).

## GPC Spectrum

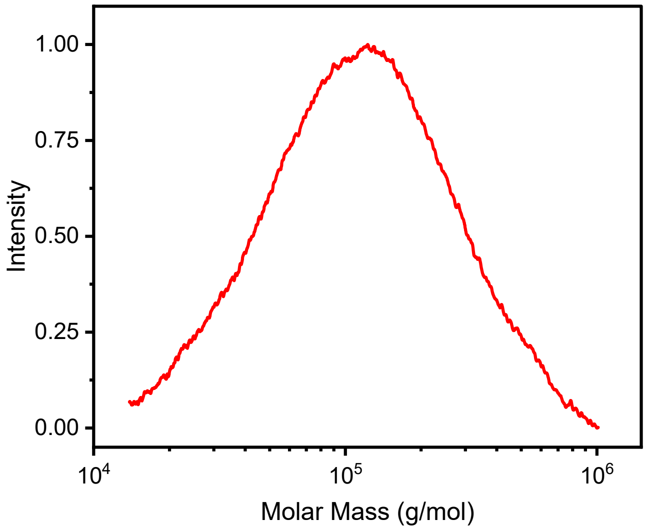


**Figure S2.** GPC elugram of poly[bis(2,2,2-trifluoroethoxy)]phosphazene.

**Table S1**. The molar mass and molar mass distributions result from GPC characterization.

| Sample | $M_n$ (g/mol) | $M_w$ (g/mol) | PDI |
|---|---|---|---|
| poly[bis(2,2,2-trifluoroethoxy)]phosphazene | 71926 | 149189 | 2.074 |

The polymer was synthesized by cationic polymerization of monomer $Cl_3PNSiMe_3$ in the presence of $PCl_5$ initiator. In general, the molecular weight is in the range of $10^5$ g/mol with a polydispersity index of 1.2 to 2.5.[1]

## FTIR Spectra

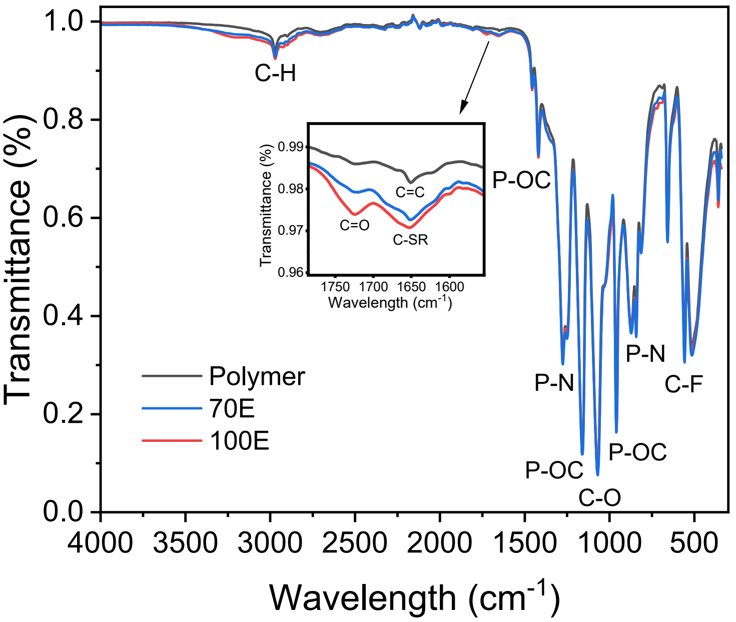


**Figure S3.** FTIR spectra of the poly[bis(2,2,2-trifluoroethoxy)]phosphazene, **E70** and **E100**.

FTIR spectra were used to determine the functional groups of the polymer and elastomers. Polyphosphazene backbone P-N-P shows an asymmetric stretching peak between 1200 and 1400 $cm^{-1}$. In general, electro-negative side groups raise the strength of the P-N bond and increase the vibrational frequency. The $OCH_2CF_3$ substituted P-N asymmetric stretching band was observed at 1270 $cm^{-1}$.[2] The symmetric P-N-P stretching appeared at 833 $cm^{-1}$. P-O-C band was observed at 1416 $cm^{-1}$, 1163 $cm^{-1}$, and 955 $cm^{-1}$.[3] The peaks at 2972 $cm^{-1}$ belong to the aliphatic C-H bond, at 573 $cm^{-1}$ belong to the C-F bond, and at 1066 $cm^{-1}$ is associated to the C-O bond stretching of the side group.[4] Additionally, the allyl peak of the polymer appears at 1650 $cm^{-1}$. Peaks corresponding to C-SR at 1653 $cm^{-1}$ and C=O at 1724 $cm^{-1}$ were observed after the thiol-ene addition reaction with the tetrakis(3-mercaptopropionate) crosslinker. No -SH peaks were detected in the region of 2600 – 2550 $cm^{-1}$, indicating that all thiol groups were consumed in the thiol-ene addition reaction.

## DSC Spectra

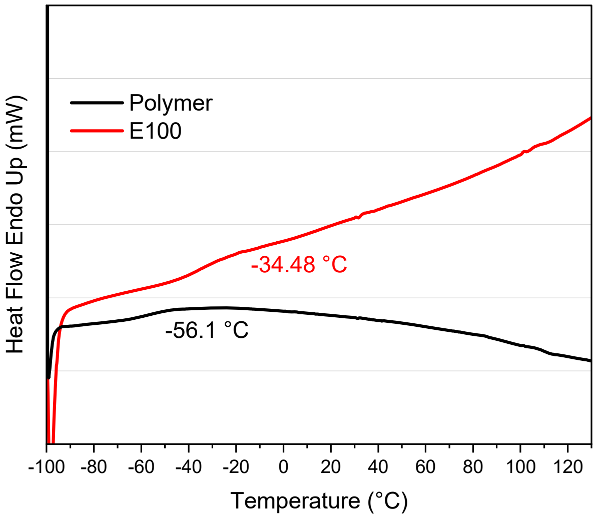


**Figure S4.** DSC graph of the poly[bis(2,2,2-trifluoroethoxy)]phosphazene, and **E100**.

Differential Scanning Calorimetry was employed to determine the glass transition temperature of both the polymer and the cross-linked material. Upon comparison, it was observed that the polymer exhibits a lower Tg than the cross-linked material, indicating reduced flexibility in the latter due to the presence of a network structure..

## TGA Spectra

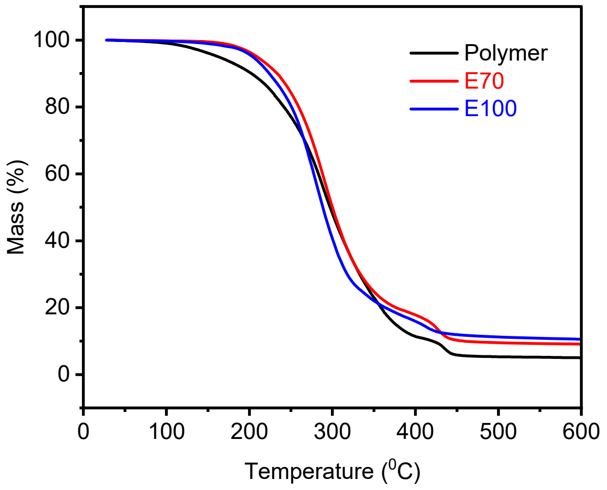


**Figure S5.** TGA graph of the poly[bis(2,2,2-trifluoroethoxy)]phosphazene, **E70** and **E100**.

Thermogravimetric analysis (TGA) was conducted using a Perkin Elmer TGA7 with a heating rate of 10 °C min⁻¹ under a nitrogen gas flow. TGA was performed to analyze the thermal stability and decomposition behavior of the polymer and the films. The analysis revealed that polymer decomposition was initiated below 160 °C. In contrast, this occurred at elevated temperatures for the elastomers, which can be attributed to the necessity of higher temperatures to disrupt the cross-linked network. The principal weight loss was observed below 350 °C due to the materials' decomposing into cyclic species.[5,6]

## Dielectric Spectra

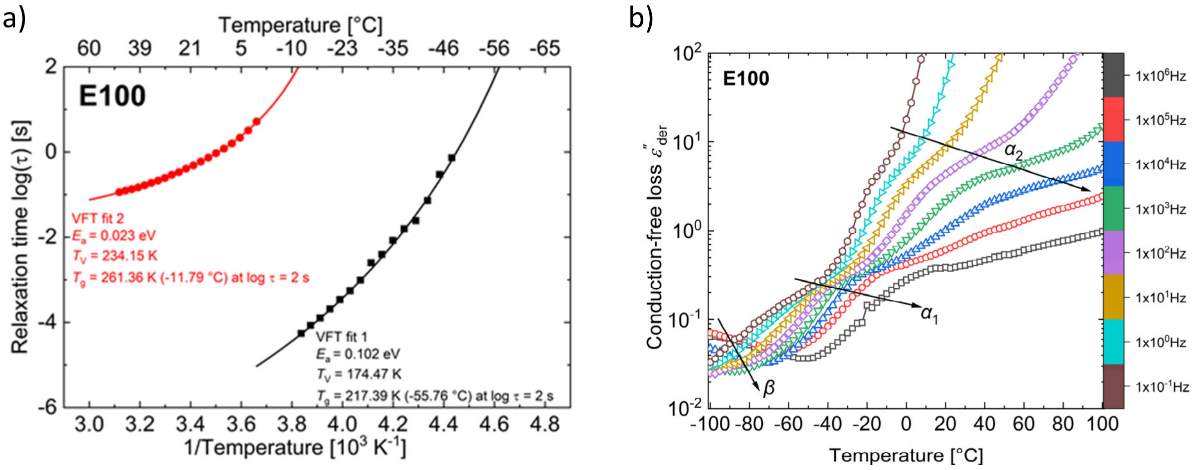


**Figure S6.** a) Arrhenius relaxation plot with VFT fits for **E100**, b) isochronal representation of the conduction-free dielectric loss ($\varepsilon''_{der}$) calculated for **E100** sample between $10^{-1}$ and $10^{6}$ Hz.

## Actuation Measurements

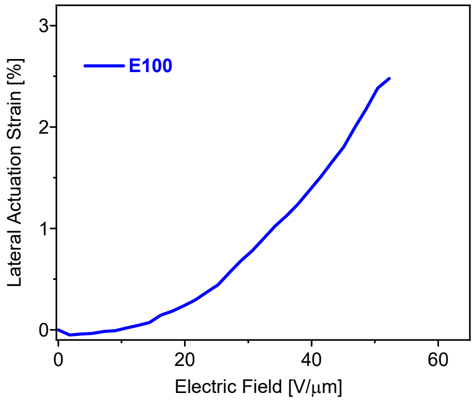


**Figure S7.** Lateral actuation strain of **E100** at different electric fields.

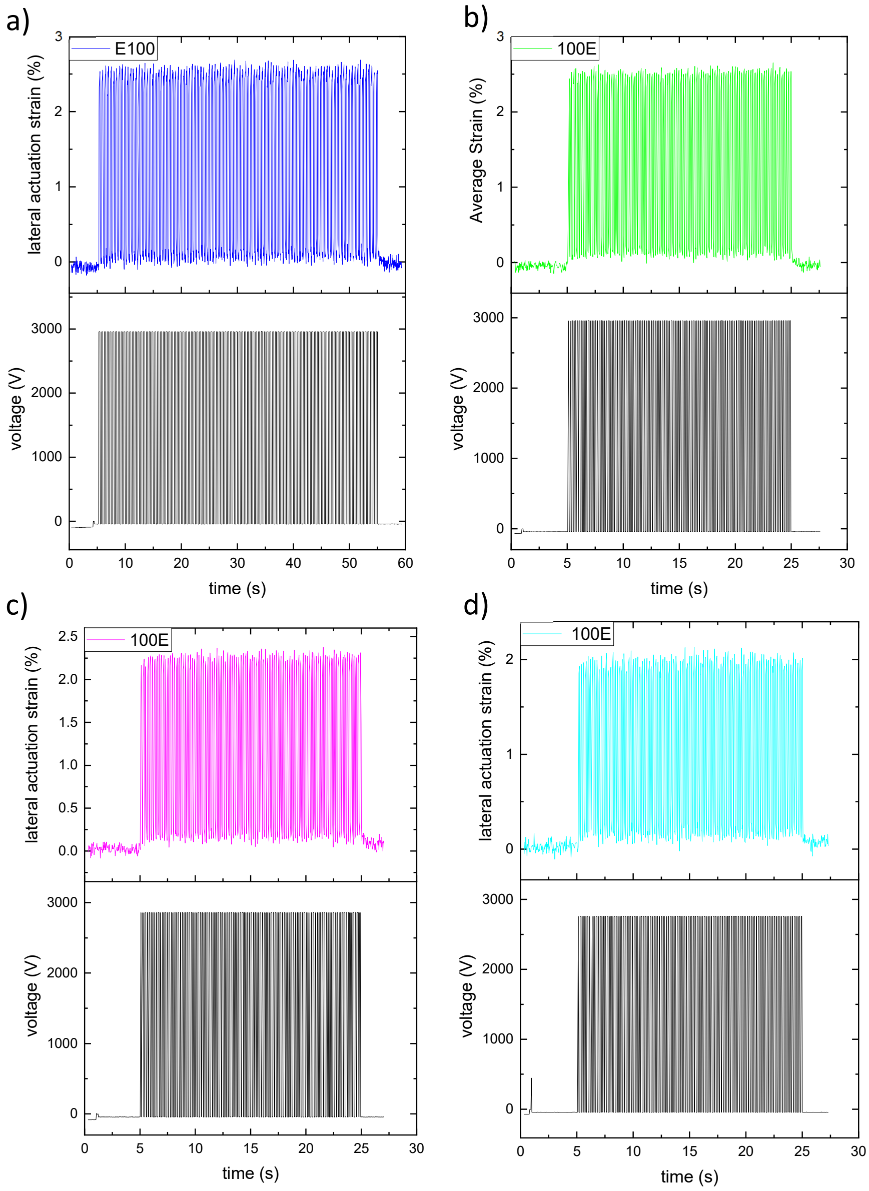


**Figure S8.** An actuator constructed from a 55 $\mu$m thick **E100** film exposed to an electric field of 52 V/µm and varying frequencies of 1, 2, 4, and 10 Hz for 100 cycles, respectively, exhibited stable and reversible actuation.

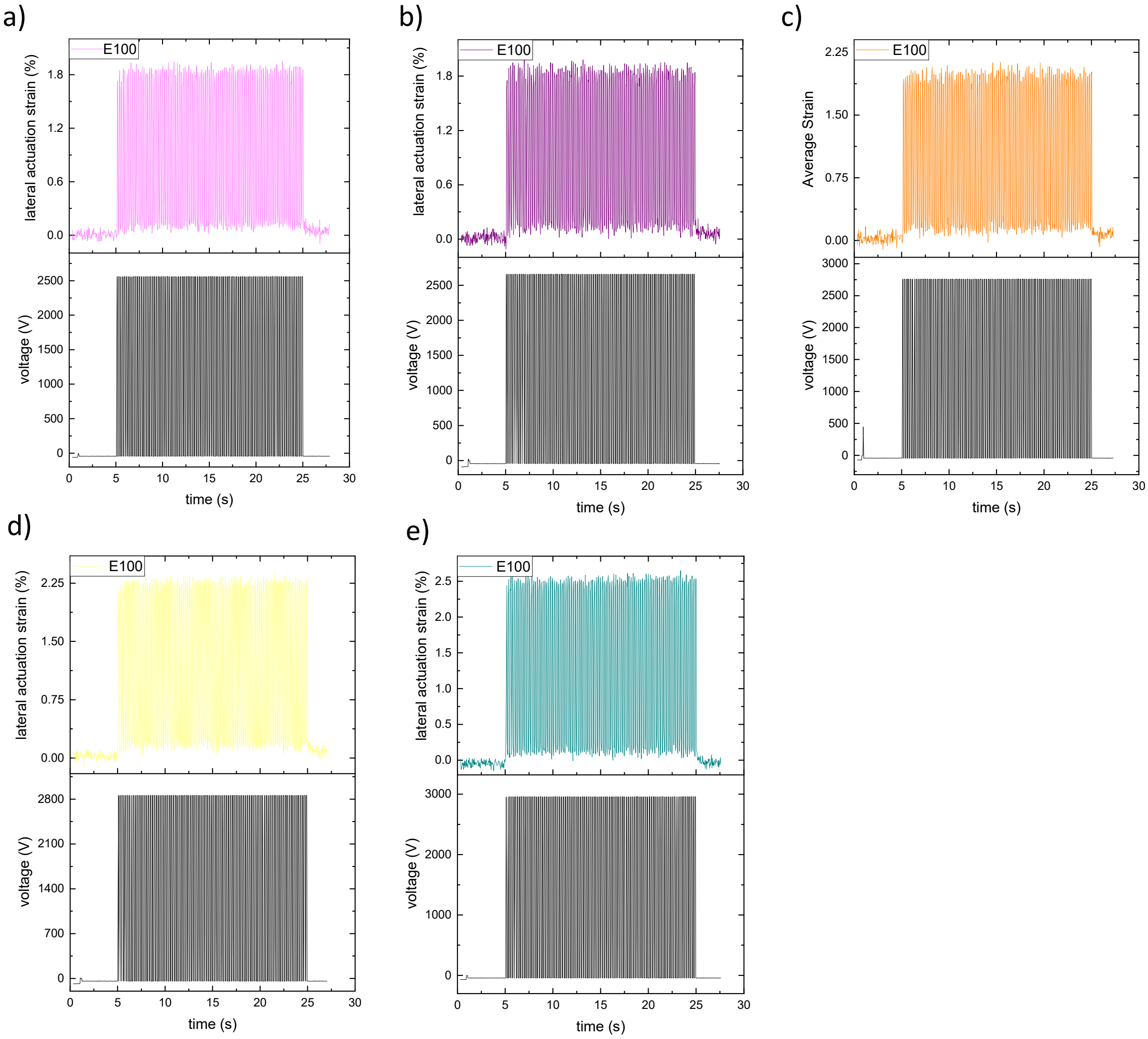


**Figure S9.** Film **100E**, 55 $\mu$m thick, subjected to different voltages of: a) 2600 V; b) 2700 V; c) 2800 V; d) 2900 V and e) 3000 V at 0.1 s for 100 cycle.

## Weibull probability plot

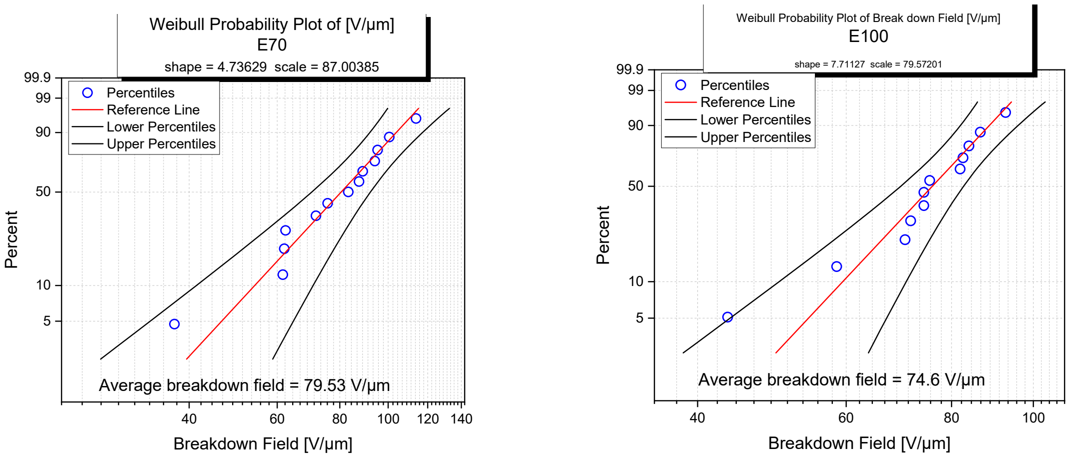


**Figure S10.** Weibull probability plot of a) **E70** and b) **E100**

## Calculation of electrostatic pressure

The electrostatic pressure acting on the films is $p = \varepsilon'\varepsilon_0 E^2$ where p is pressure, E is electric field and, ε' and $\varepsilon_0$ represents relative, and free-space ( $\varepsilon_0 = 8.854187817\times10^{-12}$ F/m) permittivity, respectively The p is calculated as:

0.32 MPa for **E70** (80 V $\mu m^{-1}$, $\varepsilon' = 5.65$) and

0.17 MPa for Elastosil (85 V $\mu m^{-1}$, $\varepsilon' = 3.00$).[7]

## References


1 H. R. Allcock, C. A. Crane, C. T. Morrissey, J. M. Nelson, S. D. Reeves, C. H. Honeyman and I. Manners, *Macromolecules*, 1996, **29**, 7740–7747.

2 R. E. Singler, N. S. Schneider and G. L. Hagnauer, *Polym. Eng. Sci.*, 1975, **15**, 321–338.

3 H. R. Allcock, R. L. Kugel and K. J. Valan, *Inorg. Chem.*, 1966, **5**, 1709–1715.

4 H. R. Allcock, J. S. Rutt and R. J. Fitzpatrick, *Chem. Mater.*, 1991, **3**, 442–449.

5 Z. Tian, A. Hess, C. R. Fellin, H. Nulwala and H. R. Allcock, *Macromolecules*, 2015, **48**, 4301–4311.

6 M. Zeldin, W. H. Jo and E. M. Pearce, *J. Polym. Sci. Polym. Chem. Ed.*, 1981, **19**, 917–923.

7 R. Pelrine, R. Kornbluh, J. Joseph, R. Heydt, Q. Pei and S. Chiba, *Mater. Sci. Eng. C*, 2000, **11**, 89–100.
